# Supplementary material for: BLADE-ON-PETIOLE proteins act in an E3 ubiquitin ligase complex to regulate PHYTOCHROME INTERACTING FACTOR 4 abundance
Source: eLife. 2017 Aug 22;6:e26759. doi: 10.7554/eLife.26759 (PMC5582868; doi:10.7554/eLife.26759)
Supplement: Figure 5—source data 1. [file elife-26759-fig5-data1.docx]

**Figure 5–source data 1 |** Quantification of ubiquitinated PIF4-HA protein levels relative to total ubiquitinated proteins from TUBEs assays.

|  | ***pif4;PIF4p::PIF4-HA*** | |  | ***pif4bop2;PIF4p::PIF4-HA*** | | |
| --- | --- | --- | --- | --- | --- | --- |
| Replicate | anti-HA | anti-Ub | Relative intensity | anti-HA | anti-Ub | Relative intensity |
| 1 | 40884.63 | 88035.22 | 0.46 | 36707.64 | 98059.11 | 0.37 |
| 2 | 45996.32 | 81282.84 | 0.57 | 35078.19 | 85174.69 | 0.41 |
| 3 | 37394.48 | 85787.47 | 0.44 | 32900.86 | 80563.70 | 0.41 |
| 4 | 29980.42 | 62707.01 | 0.48 | 38865.78 | 100423.01 | 0.39 |
| 5 | 97750.63 | 217866.79 | 0.45 | 97109.04 | 224843.91 | 0.43 |
| 6 | 52692.15 | 108456.34 | 0.49 | 53444.18 | 129110.62 | 0.41 |

Quantified data was obtained by ImageJ. The digits indicated the total pixel number of each lane.
