# Supplementary material for: BLADE-ON-PETIOLE proteins act in an E3 ubiquitin ligase complex to regulate PHYTOCHROME INTERACTING FACTOR 4 abundance
Source: eLife. 2017 Aug 22;6:e26759. doi: 10.7554/eLife.26759 (PMC5582868; doi:10.7554/eLife.26759)
Supplement: Supplementary file 1. [file elife-26759-supp1.docx]

**Supplementary file 1**

**All primer sequences used in this study.**

| **Sequence** | **Primer Name** | **Purpose** |
| --- | --- | --- |
| GGGGACAAGTTTGTACAAAAAAGCAGGCTTAATGGAACACCAAGGTTGGAG | AtPIF4_F1 | for amplification of AT2G43010(PIF4) CDS with start codon for pENTR cloning |
| GGGGACCACTTTGTACAAGAAAGCTGGGTACTAGTGGTCCAAACGAGAAC | AtPIF4_R1 | for amplification of AT2G43010(PIF4) CDS with stop codon for pENTR cloning |
| GGGGACCACTTTGTACAAGAAAGCTGGGTAGTGGTCCAAACGAGAAC | AtPIF4_R2 | for amplification of AT2G43010(PIF4) CDS without stop codon for pENTR cloning |
| GGGGACAAGTTTGTACAAAAAAGCAGGCTTAATGAGCAATCTTGAAGAATC | AtBOP2_F1 | for amplification of AT2G41370(BOP2) CDS with start codon for pENTR cloning |
| GGGGACCACTTTGTACAAGAAAGCTGGGTACTAGAAGTGATGTTGATGAT | AtBOP2_R1 | for amplification of AT2G41370(BOP2) CDS with stop codon for pENTR cloning |
| GGGGACCACTTTGTACAAGAAAGCTGGGTAGAAGTGATGTTGATGAT | AtBOP2_R2 | for amplification of AT2G41370(BOP2) CDS without stop codon for pENTR cloning |
| CTCGATTTCCGGTTATGG | SL42(pif4_L) | for genotyping *pif4_101* (GARLIC 114-G06) |
| CAGACGGTTGATCATCTG | SL43(pif4_R) | for genotyping *pif4_101* (GARLIC 114-G06) |
| GCATCTGAATTTCATAACCAATC | PD14(pif4_LB) | for genotyping *pif4_101* (GARLIC 114-G06) |
| CAATTGCCGAGGATCTTGGA | BOP2-(-708) | for genotyping SALK_075879.50.7 (*bop2-2*) |
| GTAGGTTCAGGAAATCCAACGA | BOP2-GT | for genotyping SALK_075879.50.7 (*bop2-2*) |
| TTACGAAACGCTTTTGATTCG | bop2-11_LP | for genotyping SM_3_47 |
| TATCAAGAGGCGTGATTCCAC | bop2-11_RP | for genotyping SM_3_47 |
| TCCCATCACCATTAGCTTCAC | bop1-5_LP | for genotyping SAIL_14.c02 |
| TGGTTCGGTTGAGATGAATTC | bop1-5_RP | for genotyping SAIL_14.c02 |
| TGATGCAGATTCATTCCTTCC | bop1-4_LP | for genotyping *bop1-4* GABI_386G09 |
| ACCATAGTCGCGTGAAACAAC | bop1-4_RP | for genotyping *bop1-4* GABI_386G09 |
| GCCGATGGAGATGTTGAGAT | qPIF4f | for qPCR analysis of PIF4 |
| CCAACCTAGTGGTCCAAACG | qPIF4r | for qPCR analysis of PIF4 |
| TAACGTGGCCAAAATGATGC | qPP2Af | for reference gene amplication in qPCR analysis |
| GTTCTCCACAACCGCTTGGT | qPP2Ar | for reference gene amplication in qPCR analysis |
| ATTTTGCCGATTTCGGAAC | LBb1.3 | for genotyping SALK lines |
| TAC GAA TAA GAG CGT CCA TTT TAG AGT GA | spm | for genotyping SM T-DNA lines |
| GCCTTTTCAGAAATGGATAAATAGCCTTGCTTCC | SAIL-LB1 | for genotyping SAIL lines |
| GGG CTA CAC TGA ATT GGT AGC TC | GABI_LB | for genotyping GABI T-DNA lines |
